# *Salmonella *Typhimurium-specific bacteriophage ΦSH19 and the origins of species specificity in the Vi01-like phage family

**DOI:** 10.1186/1743-422X-8-498

**Published:** 2011-11-02

**Authors:** Steven PT Hooton, Andrew R Timms, Joanna Rowsell, Ray Wilson, Ian F Connerton

**Affiliations:** 1 Division of Food Sciences, School of Biosciences, University of Nottingham, Sutton Bonington Campus, Loughborough LE12 5RD UK; 2 DeepSeq, University of Nottingham, Queens Medical Centre, Nottingham NG7 2UH UK

**Keywords:** Phage biocontrol, biosanitization, bacteriophage genomics, *Salmonella *Typhimurium, *Myoviridae*, P22-like tail spike, pectate lyase tail spike domain, lipopolysaccharide

## Abstract

**Background:**

Whole genome sequencing of bacteriophages suitable for biocontrol of pathogens in food products is a pre-requisite to any phage-based intervention procedure. Trials involving the biosanitization of *Salmonella *Typhimurium in the pig production environment identified one such candidate, ΦSH19.

**Results:**

This phage was sequenced and analysis of its 157,785 bp circular dsDNA genome revealed a number of interesting features. ΦSH19 constitutes another member of the recently-proposed *Myoviridae *Vi01-like family of phages, containing *S*. Typhi-specific Vi01 and *Shigella*-specific SboM-AG3. At the nucleotide level ΦSH19 is highly similar to phage Vi01 (80-98% pairwise identity over the length of the genome), with the major differences lying in the region associated with host-range determination. Analyses of the proteins encoded within this region by ΦSH19 revealed a cluster of three putative tail spikes. Of the three tail spikes, two have protein domains associated with the pectate lyase family of proteins (Tsp2) and P22 tail spike family (Tsp3) with the prospect that these enable *Salmonella *O antigen degradation. Tail spike proteins of Vi01 and SboM-AG3 are predicted to contain conserved right-handed parallel β-helical structures but the internal protein domains are varied allowing different host specificities.

**Conclusions:**

The addition or exchange of tail spike protein modules is a major contributor to host range determination in the Vi01-like phage family.

## Background

The use of virulent bacteriophages (phages) as biological control (biocontrol) agents against bacterial pathogens is an expanding field of research aimed at producing sustainable solutions for the control of these pathogens, and to circumvent problems such as those associated with the development of multidrug-resistant bacteria [1]. The antimicrobial activities of phages committed to the cellular lysis of a range of bacterial pathogens have been reported, which include food pathogens such as *Campylobacter jejuni *[2-5], *Escherichia coli *[6-8], and various *Salmonella enterica *serovars [4,9,10]. However, despite the wealth of data regarding the efficacy of phages during intervention studies, relatively few phage-derived products have been developed sufficiently for commercial application. Only in the last few years has there been an extension of lab-based trials into the food production environment, where perhaps the best example is the recognition of the efficacy and the granting of 'generally recognized as safe' (GRAS) status to bacteriophages targeting *Listeria monocytogenes *by the United States Food and Drug Administration [11]. There are two products of note now available commercially-ListShield™ (Intralytix Inc., USA) a phage cocktail comprising virulent phages with broad activity against *L. Monocytogenes*, and Listex P100™ (EBI Food Safety, Netherlands). Following GRAS classification both ListShield™ and Listex P100™ are now viewed as safe to be applied as food biopreservatives on ready-to-eat foods in the USA. Phage P100 (the active component of Listex P100) was initially characterized at the genetic level and in oral toxicity studies by Carlton et al [12]. These studies showed that P100 had no undesirable genes within its genome, and caused no ill effects when administered to rats. Many studies reporting the efficacy of Listex P100 against *L. monocytogenes *in various food production settings are now available in the literature [13-15].

If more phage-based applications are to reach standards where they are deemed fit for human/animal consumption then certain matters pertaining to safety must be taken into consideration. For instance, phages that show potential during preliminary studies must adhere to strict criteria if they are to be developed further as antimicrobial agents [16]. Most importantly, the selected phages must not possess genes associated with virulence, or those that may enhance the pathogenic profile of its target [17]. Many examples of phages that are recognized as being involved in such processes are known, for example, the Shiga toxin-encoding (Stx) phages-a key virulence factor of Shiga-toxigenic *E. coli *(STEC)-are the causative agents of haemolytic uraemic syndrome (HUS), a major contributor to disease associated with STEC infection [18,19]. Also, temperate phages that have the potential to form lysogens with their host need to be eliminated from trials at the earliest opportunity. Integration into, and excision out of the host genome, can lead to the transfer of genes between the phage and bacterium potentially altering the genetic profile of both [20]. These undesirable traits are most often associated with phages belonging to the *Podoviridae *(icosahedral head with a short non-contractile tail e.g. *Salmonella *phage P22) or *Siphoviridae *(icosahedral head with a flexible non-contractile tail e.g. phage λ) families. However, members of the *Myoviridae *(icosahedral head and contractile tail e.g. phage T4) are more often associated with an obligately lytic lifecycle. All of these morphological characteristics can be easily discerned when phages are viewed under a transmission electron microscope, although assumptions as to the genetic nature of a phage cannot be made on morphological characteristics alone. Whole genome sequencing of phage isolates intended for use as biocontrol agents is now considered to be the 'gold standard' in terms of transferring phage-based applications from the laboratory to everyday use.

Here we report the complete genome sequence of ΦSH19, a lytic bacteriophage adapted to infecting a number of different *S*. Typhimurium serovars. ΦSH19 has shown great potential as a biocontrol agent against *S*. Typhimurium U288, the most prevalent serovar found in UK pig production premises [21]. *S*. Typhi phage Vi01 was the first of a recently-proposed new lineage of *Myoviridae *to be described. Genetic analyses of the ΦSH19 genome reveal that it is a close relative of phage Vi01, in terms of both DNA and protein sequences. However, phage Vi01 appears to be restricted to infecting *S*. Typhi, possibly due to the presence of a virulence (Vi) capsule antigen-degrading acetyl esterase domain incorporated into one of its three tail spikes [22]. The other completely sequenced member of the Vi01-like phage lineage is SboM-AG3 that is restricted to the infection of *Shigella *spp. [23]. No genes associated with either toxicity or lysogeny have been found within the ΦSH19 157,785 bp circular dsDNA genome, nor in any of the Vi01 family of phages. A lack of virulence associated genes in ΦSH19 will allow its use as a biocontrol agent, aimed at reducing *S*. Typhimurium entering the food chain, and in particular *S*. Typhimurium U288 from pork production.

## Results

### Bacteriophage ΦSH19 characterization

**Figure 1:**
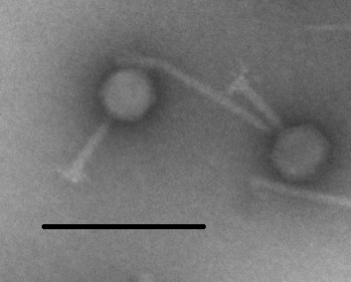
**TEM image of ΦSH19**. The presence of an icosahedral head and contractile tail indicate that ΦSH19 is a member of the *Myoviridae*. Magnification × 43,000. Bar 250 nm.

Bacteriophage ΦSH19 was originally isolated from pig intestinal contents and has specific activity against *S*. Typhimurium serovars [24]. Morphologically the phage has an icosahedral head with a contractile tail indicating it to be a member of the *Myoviridae *(Figure 1). A comparison of the lytic profiles of phages ΦSH19 and Vi01 against a panel of *S. enterica *Serovars is given in Table 1. The data shows that as expected all the *S*. Typhimurium strains tested are refractory to infection by Vi01, whereas *S*. Typhi BRD948 likewise displays immunity towards infection by ΦSH19 but susceptibility to Vi01.

**Table 1:** Host range assays of ΦSH19 and Vi01 (+ = lysis and-= no lysis).

*Salmonella*	ΦSH19	Vi01
*S*. Typhimurium U288	+	-
*S*. Typhimurium DT104 WT	+	-
*S*. Typhimurium DT104 NCTC 13348	-	-
*S*. Typhimurium WT (Rawlings)	+	-
*S*. Typhimurium WT (Turner)	+	-
*S*. Typhimurium LT2	+	-
*S*. Typhi BRD948	-	+

### Genome analysis

**Figure 2:**
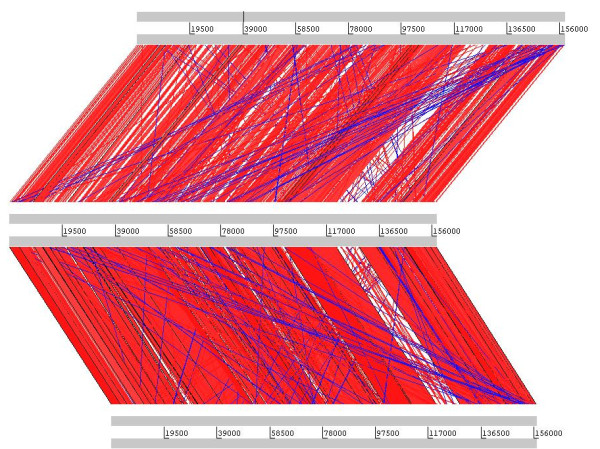
**Artemis Comparison Tool (ACT) analysis of the genomes of SboM-AG3 (top), ΦSH19 (middle), and Vi01 (bottom)**.

The ΦSH19 genome was sequenced from sonicated DNA fragments using the Roche 454 GS FLX platform (17,796 reads), from which a single contig of 157,785 bp was generated. BlastN analysis of the ΦSH19 genome revealed two related genome sequences in the database-phages Vi01 (Genbank Acc. No. FQ312032) and SboM-AG3 (Genbank Acc. No. FJ373894). The ΦSH19 genome was ordered such that it could be directly aligned with the existing genomes of Vi01 and SboM-AG3 commencing with the *rIIA *gene. The GC-content of the ΦSH19 genome was determined to be 44.68%, which is slightly lower than Vi01 (45.22% GC), and considerably lower than SboM-AG3 (50.39% GC). Alignment of the nucleotide sequences of ΦSH19 and Vi01 indicate that they are highly similar over a large proportion of the genome (between 80-98% pairwise identity). However, for a region located between 120-130 Kb on the genome map this homology breaks down to approximately 70% identity (and in some instances the conservation is lost completely). This region contains a cluster of three putative tail spike reading frames in all of the Vi01-like phages sequenced to date. At the nucleotide level ΦSH19 is somewhat less similar when compared to SboM-AG3, with homologies ranging from 74-100% identity over approximately 60% of the genome. Comparison of the varying degrees of homology between ΦSH19, Vi01, and SboM-AG3 are represented in Figure 2. Otherwise highly similar nucleotide matches were restricted to components of *Escherichia *phage PhaxI (Genbank Acc. No. HQ259289), *Serratia *phage KSP90 (Genbank Acc. No. AB452990) and *Salmonella *phage Det7 (Genbank Acc. No. AM765843).

**Figure 3:**
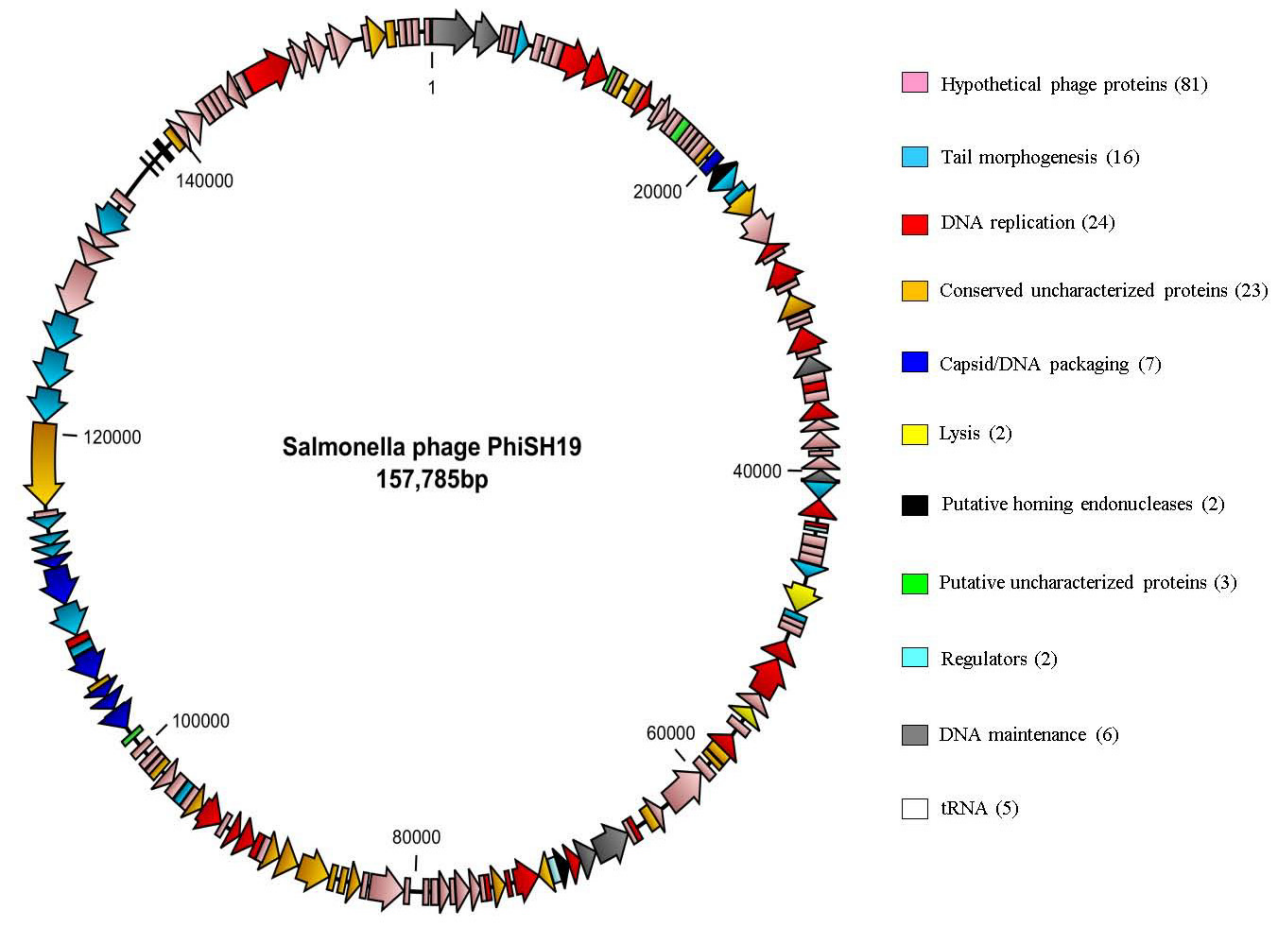
**A schematic circular map of the ΦSH19 genome**.

Initial analysis of the ΦSH19 genome identified 339 potential open reading frames (ORFs). Following BlastP and Pfam domain analyses of each potential ORF, the candidate ORFs were reduced to 166. Each ORF was then annotated using Vi01 and SboM-AG3 phages as reference sequences, and categorised as follows: Hypothetical phage proteins (81), Conserved hypothetical phage proteins (23), DNA replication (24), Tail morphogenesis (16), Capsid/DNA packaging (7), DNA maintenance/circularization (6), Putative uncharacterized proteins (3), Regulators (2), Lysis (2), and Putative homing endonucleases (2). A comparison of the major proteins of ΦSH19 with their homologues in Vi01 and SboM-AG3 is presented in Table 2. Figure 3 shows the fully annotated ΦSH19 genome as a circular genetic map. Using tRNAscan-SE, five tRNA genes were also identified in a 5 Kb non-coding region of the ΦSH19 genome at the following nucleotide positions: Methionine tRNA (CAT) 137,703-137,735; Asparagine tRNA (GTT) 137,806-137,878; Tyrosine tRNA (GTA) 138,497-138,577; Serine tRNA (GCT) 138,657-138,742; and an undetermined tRNA 138,749-138,834. For phages ΦSH19, Vi01, and SboM-AG3 there exists a set of tRNA genes located at similar positions in their respective genomes, some of which display a high degree of conservation. For example, BlastN analysis of the coding sequence for tRNA-Serine that is present in all three phages indicates 100% identity at the nucleotide level. Two other tRNAs (tRNA-Asparagine and tRNA-Tyrosine) are also shared between the group. For ΦSH19 tRNA-Asparagine, the gene is 100% identical with its homologue in Vi01 and 95% identical to that of SboM-AG3, whilst the ΦSH19 tRNA-Tyrosine shows more conservation with that of SboM-AG3 (99% identical) than Vi01 (84% identical). ΦSH19 also shares a tRNA-Methionine gene (88% identity) and a tRNA of undetermined specificity (99% identity) with Vi01 that are absent in SboM-AG3. Phire analysis for phage regulatory elements identified a number of sites in the ΦSH19 genome (Table 3). All of the putative regulatory elements are found on the non-coding strand (relative to the transcribed sequences within that region) however they appear to be associated with ORFs on the opposite strand. It is quite possible that these elements play a role in regulating ΦSH19 gene expression, in an as yet undetermined manner, but perhaps by the production of small RNA molecules.

**Table 2:** Comparison of ΦSH19 proteins with those of Vi01 and SboM-AG3

CDS	ΦSH19 coordinates & amino acid length	Predicted MW	Pfam domain	Amino acid ID with Vi01	Amino acid ID with SboM-AG3
RIIA	1-2742 (913)	104,992	None	98%	54%
RIIB	2775-4358 (527)	58,980	None	97%	69%
Putative tail fibre	5502-6311 (269)	27,826	Ig-like I-set domain (CL0011)	86%	86%
DNA topoisomerase II	8523-10,436 (637)	71,862	HATPase c (CL0025) & DNA gyrase B	96%	90%
DNA topoisomerase II (medium subunit)	10,357-11,769 (470)	53,939	DNA topoisomerase IV	88%	88%
Conserved uncharacterized protein	13,309-13,929 (206)	23,005	Putative Macro domain of ADP-ribose binding module (CL0233)	92%	74%
DexA exonuclease	14,263-14,895 (210)	24,438	None	95%	93%
dCMP deaminase	18,704-19,225 (173)	19,460	dCMP cytosine deaminase 1 family-cytidine & deoxycytidylate deaminase Zn-binding domain (CL0109)	97%	62%
Conserved uncharacterized protein	19,227-19,634 (135)	15,378	Bacterial membrane-flanked domain (DUF304)	93%	88%
Head completion protein	19,845-20,465c (206)	24,189	None	92%	74%
Putative homing endonuclease	20,465-21,205c (246)	28,205	GIY-YIG catalytic domain (CL0418)	No match	34%
T4-like baseplate tail tube cap	21,259-22,227 (322)	36,142	T4 tail cap family	100%	94%
Baseplate wedge subunit	22,240-22,797 (185)	21,574	Phage Gp53 family	97%	80%
Loader of T4-like helicase	26,134-26,796c (220)	26,253	T4 helicase N family	99%	79%
DNA ligase	27,281-28,708c (475)	53,501	ATP-dependent DNA ligase domain (CL0078)	95%	83%
DNA primase-helicase subunit	31,451-32,875c (474)	54,192	DnaB-like helicase N & C terminal domains	98%	88%
UvsX (RecA-like protein)	33,189-34,274c (361)	40,791	RecA bacterial DNA recombination proteins (CL0023)	72%	78%
dUTPase	34,789-35,340c (183)	21,114	dUTPase 2 family(CL0231)	69%	63%
Thymidylate synthase	35,903-36,943c (346)	39,216	Thymidylate synthase family	93%	77%
DNA end protector	39,924-40,268c (234)	28,347	None	100%	91%
Baseplate tail tube	40,488-41,624 (378)	42,658	Phage T4 Gp19 family	98%	90%
ssDNA binding protein	41,652-42,683c (343)	38,896	Gp3 DNA binding protein-like domain	98%	77%
Late promoter transcription factor	43,029-43,331c (100)	11,175	None	100%	70%
Regulatory protein (FmdB family)	43,264-43,509c (81)	8732	CXXC CXXC SSSS family containing a Zn ribbon domain (CL0167)	96%	88%
Putative uncharacterized protein	43,875-44,396c (203)	23,739	RuvC family (crossover junction endoribonuclease RuvC)	100%	83%
Baseplate hub subunit	45,471-46,274 (267)	30,186	T4 baseplate family	96%	67%
Tail-associated lysozyme	46,781-48,406 (541)	59,023	Gp5 OB family & CHAP domain (CL0125) associated with peptidoglycan hydrolysis	98%	87%
Baseplate wedge protein	48,471-48,851 (126)	14,124	GPW Gp25 family (gene 25-like lysozyme)	97%	87%
NrdB	50,229-51,332c (367)	42,209	Ribonucleotide reductase small chain domain (CL0044)	99%	89%
NrdA	51,403-53,730c (775)	88,137	ATP-cone domain, ribonucleotide reductase IgN all alpha domain & IgC barrel domain	94%	89%
PhoH-like phosphate starvation-inducible protein	53,764-54,603c (279)	31,567	PhoH-like protein family (CL0023)	100%	88%
Peptidoglycan binding protein	54,711-55,505c (264)	28,815	Peptidoglycan binding protein domain (CL0244) & protein of unknown function (DUF3380)	98%	86%
DNA primase subunit	56,712-57,776c (354)	41,386	None	97%	77%
Conserved uncharacterized protein	63,656-64,273c (205)	22,902	T4 RegB endoribonuclease family (CL0037)	98%	79%
NrdA.1	64,978-65,313c (336)	12,934	None	94%	92%
Recombination endonuclease subunit	65,622-67,955c (777)	88,531	None	98%	72%
Recombination protein subunit	67,958-69,073c (371)	43,030	None	98%	91%
σ factor for late transcription	69,060-69,854c (264)	30,775	None	90%	77%
Putative homing endonuclease	69,854-70,576c (240)	27,188	None	No match	36%
RNase H	70,587-71,114c (175)	19,878	RNase H domain (CL0219)	97%	71%
ATP-dependent helicase	71,921-73,522c (533)	60,690	None	99%	86%
DNA binding protein	73,778-74,056c (92)	9728	Bacterial DNA-binding protein domain	100%	86%
Conserved uncharacterized protein	74,140-74,931c (263)	28,556	SPFH domain/Band 7 family (CL0433)	98%	No match
Superinfection exclusion protein	75,137-75,457c (106)	12,562	None	95%	No match
ImpD	75,736-76,407c (223)	25,665	None	95%	74%
Acyl carrier protein	80,335-80,687c (110)	12,162	Phosphoantetheine (PP)-binding family (CL0314)	92%	61%
Conserved uncharacterized protein	84,506-84,925c (139)	15,622	Protein of unknown function (DUF3268)	90%	84%
Putative RegA translational repressor	89,926-90,390c (154)	18,078	Bacteriophage translational regulator	99%	90%
Clamp loader for DNA polymerase	90,420-90,842c (140)	16,200	None	98%	76%
Gp44 sliding clamp holder	90,847-91,836c (329)	37,214	ATPase family (CL0023)	99%	87%
Gp45 sliding clamp holder	91,914-92,582c (222)	24,510	Gp45 sliding clamp C terminal	99%	82%
Putative type III restriction enzyme (RE)	93,293-94,792 (499)	57,802	Type III RE subunit (CL0023) & helicase conserved C terminal domain (CL0023)	95%	81%
Conserved uncharacterized protein	94,822-95,568c (248)	28,911	PD-(D/E)XK nuclease superfamily	97%	86%
Putative UvsY	95,568-96,023c (151)	17,991	UvsY protein family (recombination, repair, & ssDNA binding protein	93%	85%
Tail completion protein	96,062-96,559c (165)	18,542	T4 Gp19 family	98%	80%
Major capsid protein	101,588-102,910c (440)	48,059	Gp23 major capsid protein family	98%	94%
Prohead core scaffold protein	103,002-103,841c (279)	30,898	None	96%	74%
Prohead protease	103,888-104,556c (222)	24,482	Peptidase U9 family (CL0201)	99%	92%
Portal vertex protein of the head	105,102-106,784c (560)	63,094	T4 Gp20 family	99%	81%
Tail tube protein	106,852-107,385c (177)	19,912	T4 Gp19 family	100%	98%
GIY-YIG endonuclease	107,416-107,874c (152)	17,316	GIY-YIG catalytic domain endonuclease family (CL0418)	95%	32%
Tail sheath protein	107,933-109,828c (631)	68,439	Phage sheath 1 family	98%	92%
Large terminase subunit	109,881-112,091c (736)	84,547	Terminase 6 family	95%	85%
Small terminase subunit	112,072-112,752c (226)	24,825	DNA packaging family (terminase DNA packaging enzyme)	98%	74%
Proximal tail sheath stabilization	112,755-113,453c (232)	26,979	None	98%	80%
Gp14 neck protein	113,456-114,097c (213)	24,759	T4 neck protein family	100%	82%
Gp13 neck protein	114,400-115,209c (269)	31,142	None	99%	87%
Tail spike 1	120,710-122,641c (643)	68,851	None	67% (res. 1-171 only)	62% (res. 1-190 only)
Tail spike 2	122,702-124,876c (724)	78,194	Pectate lyase C (CL0268)	86% (res. 1-276 only)	73% (res. 1-161 only)
Tail spike 3	124,992-127,088c (698)	75,785	P22 tail spike family	86% (res. 1-154 only)	46% (res. 1-179 only)
Haemolysin-type calcium binding protein	127,168-130,209c (1013)	108,436	None	81% (res. 1-418 only)	63% (res. 1-404 only)
Baseplate wedge subunit	132,287-134,080c (597)	66,068	None	98%	79%
DNA polymerase	145,442-148,438 (998)	116,583	DNA polymerase family B exonuclease domains (CL0194 & CL0219)	98%	86%
Putative uncharacterized protein	148,776-149,615 (279)	32,346	NT5C family 5' nucleotidase deoxypyrimidine (CL0137)	97%	80%

**Table 3:** Putative phage regulatory elements identified by Phire analysis.

Putative phage regulatory elements		
**From**	**To**	**Sequence**

56,300	56,319	TAGCCATGATGTAATTCCTC
79,926	79,945	CGGTCATGATGTATTTCCTT
99,487	99,506	TGGTCATGGTGTAGTTCCTT
101,431	101,450	GTCCCATGATGTATTTCTTC
104,872	104,891	TTTCCATCATGCATCTCCTT
109,822	109,841	TTGCCATAATATCATTCCTT
144,434	144,453	TTGCCATGATGTATTTCCTT

### ΦSH19 tail spikes

Three tail spike genes-*tsp1 *(bases 120,710-122,641c), *tsp2 *(122,702-124,876c), and *tsp3 *(124,992-127,088c) were identified during the annotation of ΦSH19. BlastN analysis of the *tsp1 *gene (1932 bp) shows that the nucleotide sequence has 94% identity with that of Vi01 orf 170c (Vi01 Tsp1) over the first 263 bases, but beyond this conservation breaks down and no further nucleotide identities are observed between the two genes. A similar sequence to *tsp1 *is present at orf 00207 (SboM-AG3 Tsp1) of SboM-AG3 (64% identity over the first 390 bases), and a shorter stretch of homology (78% identity over the first 54 bases) is located within orf 00212 (SboM-AG3 Tsp3). As with Vi01, no further identity can be observed with *tsp1 *following these short stretches of conservation. Other nucleotide alignments of *tsp1 *include identities with the *Salmonella *phage Det7 tail spike gene (86% identity from bases 360-460), and an endo-α-sialidase gene of coliphage K1F (76% identical from bases 351-485). Aside from these alignments, which all span a region covering the first 485 bases of *tsp1*, no homologies were found in the database for the region covering bases 485-1932. Alignment of the *tsp2 *gene (2175 bp) reveals that only Vi01 and SboM-AG3 have similar sequences within the database. As with *tsp1*, the homologies all span the start of the gene: 85% identity (bases 1-723) with Vi01 orf 171c (Vi01 Tsp2-maturation/adhesion protein) and 70% identity (bases 1-360) with SboM-AG3 orf 00210 (SboM-AG3 Tsp2). The latter portion of the *tsp2 *gene does not align with any sequences in the database. BlastN searches involving the *tsp3 *gene (2097 bp) show that the sequence is highly similar to the Det7 tail spike gene (85% identity from bases 1-782). To a lesser extent, *tsp3 *shares sequence homology (84% identity between bases 1-461) with Vi01 orf 172c (Vi01 Tsp3) and orf 170c (69% identity between bases 1-179). Apart from these short regions of homology, there are no further identities between *tsp3 *and Vi01. Interestingly, no nucleotide sequence homology was found between *tsp3 *and SboM-AG3. Bases 481-667 show a conserved sequence that is shared between several phage genomes in the database. Sequence alignments over this region indicate 74-77% shared identities between various *Salmonella *phages including P22, ST104, ST64T, ST160, SE1, and some of the *S*. Enteritidis typing phages (SETP) [25]. Interestingly, the sequence is also present in prophage tail spikes of *S*. Typhimurium D23580, *S*. Typhimurium T000240, *S*. Heidelberg SL476, and *S*. Paratyphi A (strain AKU 12601). As was the case for *tsp1 *and *tsp2 *no homologous sequences are found to match the latter region of the *tsp3 *gene.

**Figure 4:**
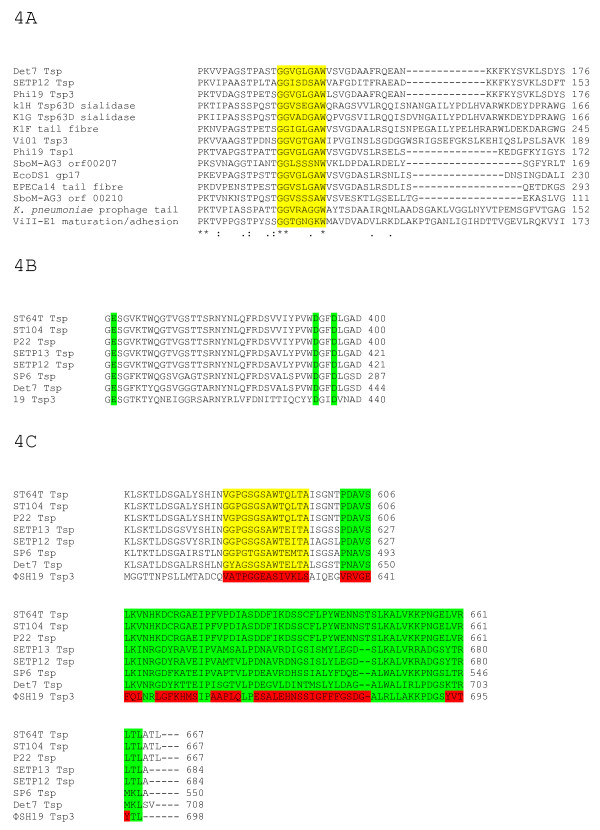
**A-C ClustalW2 alignments of sequence motifs in tail-associated proteins (4A), the active site of P22-like tail spikes (4B), and the C-terminal region of P22-like tail spikes-yellow-sequence motif (residues 585-596 in P22) associated with intertwined region, green-five-stranded β-sheet and three-stranded β-sheet (residues 601-664 in P22), and red-regions lacking homology in ΦSH19 Tsp3 (4C)**.

Translation of the *tsp *gene sequences indicate that Tsp1 is a 643 amino acid protein (predicted MW ~ 68.9 kDa), Tsp2 is comprised of 724 amino acids (predicted MW ~ 78.2 kDa), and Tsp3 698 amino acids (predicted MW ~ 75.8 kDa). Pfam domain searches for each tail spike indicated significant domain matches for Tsp2 (Pectate lyase domain-family 3 CL0268) and Tsp3 (P22 tail spike family); however no significant domain similarities were identified for Tsp1. Betawrap analyses of the amino acid sequences of each tail spike from ΦSH19, Vi01, and SboM-AG3 shows that each of these proteins potentially have the ability to form right-handed parallel β-helical structures. A comparison of each ΦSH19 tail spike protein sequence with those of Vi01 and SboM-AG3, and other homologous proteins in the database revealed the N-terminal residues (1-130) of ΦSH19 Tsp1 are highly conserved with the corresponding regions in Vi01 Tsp1 (78% ID), SboM-AG3 Tsp1 (75% ID), and to a lesser degree in Vi01 Tsp3 (56% ID). This N-terminal homology is also shared with the *Salmonella *phage Det7 tail spike (56% ID), and a tail fibre protein from *Enterobacter *phage Ecp1 (42% ID). A sequence motif present in ΦSH19 Tsp1 G-G-V-G-L-G-A-W (beginning at residue 143) possibly signifies the boundary at which the catalytic domain of the tail spike begins. No such sequence motif is present in Vi01 Tsp1, however similar sequence motifs are present at analogous positions within SboM-AG3 Tsp1 (G-G-L-S-S-S-N-W) and Vi01 Tsp3 (G-G-V-G-T-G-A-W). ClustalW2 alignments of phage tail-associated proteins containing derivatives of these motifs at their N-terminal boundaries are shown in Figure 4A. The C-terminus of ΦSH19 Tsp1 (residues 300-643) produced no significant alignments with any known proteins. BlastP analyses of Vi01 Tsp1 and SboM-AG3 Tsp1 was performed to identify any functionally-related proteins. However, as with ΦSH19 Tsp1, Vi01 Tsp1 produced no significant alignments over the C-terminal region with any protein sequences in the database.

Alignment of ΦSH19 Tsp2 with Vi01 Tsp2 (maturation/adhesion protein) and SboM-AG3 Tsp2 shows a high degree of conservation from residues 1-160. At this point SboM-AG3 sequence homology (73% ID over residues 1-162) breaks down with no further identity to either Vi01 or ΦSH19 Tsp2. Residues 100-403 in ΦSH19 Tsp2 show a weak but significant relationship with a phage structural protein/putative tail fibre (orf 00213) present in SboM-AG3. For ΦSH19 Tsp2 and Vi01 Tsp2, homology continues over the first 276 residues (86% ID) at which point the two sequences completely diverge from each other. Another region of low homology (residues 100-316) is also found with a putative tail fibre protein encoded in the Vi01 genome (Vi01 orf 173c). A pectate lyase protein family domain (family 3-CL0268) spanning residues 291-549 was identified during Pfam analysis of ΦSH19 Tsp2. The presence of this motif was further confirmed during BlastP analysis, with a number of alignments being made with various glycoside degrading enzymes from a wide range of microorganisms. No significant alignments with the C-terminus of ΦSH19 Tsp2 were identified (residues 557-724).

ΦSH19 Tsp3 alignment with Vi01 Tsp3 shows a high degree of sequence conservation between their N-terminal residues 1-154 (86% ID). However, the corresponding sequence in SboM-AG3 Tsp3 shows similarity over the first 19 residues before a deletion of 63 amino acids, after which the homology is restored between the three tail spike sequences. Interestingly, SboM-AG3 Tsp1 shows a higher degree of conservation with ΦSH19 Tsp3 over residues 1-139 (46% ID) than does SboM-AG3 Tsp3. The boundary motif G-G-V-G-L-G-A-W appears in ΦSH19 Tsp3 at residues 143-150 with analogous sequences in Vi01 (G-G-V-G-T-G-A-W) and SboM-AG3 (G-G-V-S-S-S-A-W), after which the sequence homologies between all three tail spikes break down. Pfam domain analysis of ΦSH19 Tsp3 identified a protein domain (residues 159-698) with significant homology to the P22 tail spike family (PF09251). BlastP analysis produced alignments with Det7 (the first reported Myovirus with a podoviral tail spike), P22, and a number of P22-like phages. The highest degree of homology was found for the tail spike of Det7 (77% ID over the first 300 residues). The sequence motif marking the domain boundary in ΦSH19 Tsp3 (G-G-V-G-L-G-A-W) is conserved in Det7. P22 tail spike-like sequences feature three conserved residues that function as catalytic components of the endo-rhamnosidase activity associated with P22 tail spike protein [26,27]. These residues are located in the substrate binding cleft of the P22 tail spike and are conserved in ΦSH19 Tsp3 (Figure 4B: Glu-359, Asp-392 and Asp-395).

## Discussion

Bacteriophage ΦSH19, a candidate for use as a biocontrol agent against *Salmonella *Typhimurium, was subjected to whole genome sequencing. This process is now viewed as a pre-requisite to using phages as therapeutic agents, especially if they are to be introduced into a food production environment. The presence or absence of genes associated with toxicity or lysogeny will ultimately govern whether or not a potential phage candidate is safe for commercial use. ΦSH19 was found to contain no undesirable genes in its 157,785 bp genome making it an excellent candidate for such applications. Also, ΦSH19 constitutes a new addition to the Vi01-like phage family (along with *S*. Typhi phage Vi01 and *Shigella *phage SboM-AG3). In many respects ΦSH19 is quite similar to Vi01 with the addition of a putative homing endonuclease and a putative uncharacterized protein. Structural genes and those associated with morphology of the phage particle are highly conserved between ΦSH19 and Vi01, and to a lesser degree with SboM-AG3. Analysis of the tRNA genes encoded by all three phages, and their location within each genome indicates that some of them are possibly derived from a common ancestor. Transmission electron microscope images of ΦSH19 (Figure 1), Vi01 and SboM-AG3 indicate that their morphologies are quite similar. The intricate 'chandelier-like' arrangements of tail spikes attached to the baseplate are visible for all three phages [22,23]. It seems likely that there are more Vi01-like phages (such as *Escherichia *phage PhaxI) capable of infecting a diverse range of bacterial pathogens to be discovered for which there are commercial applications.

The modular structure of the three tail spikes found in ΦSH19 is potentially the main driving factor behind host-range determination for this phage. Although no function can at present be assigned to Tsp1, the other two tail spikes contain defined protein domains that provide evidence as to their mode of action. For Tsp2, the pectate lyase domain indicates that this protein may well function to modify or cleave glycoside bonds. The pectate lyase family domain (CL0268) notably contains proteins with pectate/pectin lyase and pectin methylesterase activities. The pectate/pectin lyases are a well-characterized family of proteins principally involved in microbial plant pathogenesis. Their primary mode of action involves the eliminative cleavage of α-1,4 linked galacturonosyl residues of pectins that are components of the middle lamella of plant cell walls. The three dimensional structure of *Erwinia chrysanthemi *EC16 pectate lyase C (PelC) was first solved by Yoder et al. [28] and is representative of a family of proteins containing right-handed parallel β-helical structures with the flexibility to allow protein loops from the stacked coils to add functionality, for example the formation of the active site clefts that support enzyme catalysis [29,30]. Using a similar base architecture pectin methylesterase acts to de-esterify pectin to pectate. Members of the pectinolytic protein family are represented in prokaryotic and eukaryotic microorganisms, and also function in plants to remodel cell walls [29]. Divergence from the ancestral sequence over time has allowed different microorganisms to target a range of pectin-like substrates, while the overall structure has been maintained [30]. It is of little surprise that phages have evolved the ability to acquire these proteins and utilize their polysaccharide modifying properties. In the case of ΦSH19 Tsp2 it could be postulated that the target is the α-D-galactose (1-4) α-D-mannose linkage found in the *S*. Typhimurium O-antigen trisaccharide repeat. Many phage tail-associated proteins have been found to contain right-handed parallel β-helical structural domains, for example phages P22 [31], Sf6 [32], HK620 [33] and Det7 [27]. Moreover the presence of a pectate lyase-type structure in a phage tail spike protein has previously been reported for K5 lyase (KflA) of coliphages K5A and K1-5. KflA targets and degrades the capsular polysaccharide of *E. coli *K5, thus allowing the phage access to outer membrane receptors [34]. Whilst the precise identity of the polysaccharide target of ΦSH19 Tsp2 is at present unknown, it is possible that this tail spike plays a major role in host-range determination. Plaque assays using phages Vi01 and ΦSH19 against a panel of *S*. Typhimurium and *S*. Typhi BRD948 indicate that these phages are specific for their host (Table 1). This is perhaps not surprising since Vi01 orf 171c (Vi01 Tsp2) possesses a 9-0-acetyl esterase/acetylxylan esterase domain (residues 343-445; DUF303) that targets the acetyl-modification of the *S*. Typhi Vi capsule [22]. The pectate lyase domain found in ΦSH19 Tsp2 is more than likely involved in the degradation of a polysaccharide present on the surface of *S*. Typhimurium U288. Anany et al. [23] also report that SboM-AG3 is *Shigella*-specific and is unable to form plaques on lawns of any of the *Salmonella*, *Escherichia*, and *Listeria *strains tested.

A protein domain composed of parallel right-handed β-helices is also found in ΦSH19 Tsp3-the P22 tail spike domain. Many tail spike proteins possess an N-terminal binding domain involved in attaching the protein to the virion head/tail structure (the sequence of which is often highly conserved between phages with similar morphologies), a central catalytic domain (containing the trimeric parallel right-handed β-helices), and a C-terminal trimerization domain (involved in stabilization of the trimeric tail spike). The trimeric β-helices that form the catalytic domain bind and cleave polysaccharides present in bacterial lipopolysaccharide (LPS). P22 tail spike (Gp9) utilizes this endo-rhamnosidase activity to degrade the *Salmonella *O antigen [35]. Cleavage of α-L-rhamnose (1-3) α-D-galactose found in the trisaccharide-repeats of *S*. Typhimurium LPS takes place on the external surface of the tail spike. The structure and arrangement of the parallel β-helix tail spike domains result in a solvent-exposed exterior that features the proposed catalytic residues Glu-359, Asp-392, and Asp-395 [27,36]. The active site residues and their sequence environments are conserved in ΦSH19 Tsp3, and therefore can be postulated to operate in a manner akin to the P22 tail spike and its relatives. Thus, there is a possibility that ΦSH19 Tsp3 acts in two distinct ways. Firstly, ΦSH19 Tsp3 may function as an initial step towards irreversible adsorption of the phage particle to its host, hydrolyzing the outer *S*. Typhimurium LPS layer in order to provide access to a previously inaccessible outer membrane receptor. Secondly, the endo-rhamnosidase activity associated with P22-like tail spikes may aid phage progeny in freeing themselves from cellular debris encountered during host lysis [37].

Tsp3 of ΦSH19 has an N-terminal sequence similar to Vi01 orf 172c (Vi01 Tsp3), SboM-AG3 orf 00207 (SboM-AG3 Tsp1) and Det7 tail spike. These phages are all *Myoviridae *of similar morphologies. Therefore the requirements of having to attach the tail spike to similar base plate structures more than likely places constraints on sequence divergence. Likewise, the alignments for the *Podoviridae *tail spikes show N-terminal conservation between related phage, followed by conserved catalytic and C-terminal domains that are shared between *Myoviridae*, *Podoviridae *and *Siphoviridae*. Interestingly, P22, ST64T, and ST104 as members of the *Podoviridae *are 98-99% identical over their 667 amino acid tail spike proteins, and similarly *Siphoviridae *members SETP12 and SETP13 also have almost identical tail spike sequences. Despite that the SETP 12 and 13 phage sequences are markedly different from P22, ST64T, and ST104 over the N-terminal regions; however, they become conserved between residues 140 to 684. As noted above, there appears to be sequence divergence constraints placed on conserved N-terminal regions associated with the myoviruses ΦSH19, Vi01, SboM-AG3 and Det7. Similarly it appears there are structural restrictions imposed on the *Podoviridae *and *Siphoviridae *N-terminal tail spike sequences. Similarities between ΦSH19 Tsp3, Det7 and the P22-like sequences begin ten amino acids after the boundary motif G-G-V-G-L-G-A-W that appears in ΦSH19 Tsp3 and Det7 (this motif also signals the divergence of the tail spike sequences of phages Vi01 and SboM-AG3 from ΦSH19 and Det7). ΦSH19 Tsp3 and Det7 Tsp show striking conservation with the P22-like sequences over the catalytic domain, up to and including the active site residues Glu-359, Asp-392 and Asp-395. However, immediately after the active site in ΦSH19 Tsp3 (residues 440-698) sequence conservation breaks down.

For P22-like tail spike proteins, the C-terminal residues 585-596 form an intertwined region that allows extensive hydrogen bonding between subunits, whilst residues 606-664 form a five-stranded and a three-stranded β-sheet region [27]. ClustalW2 alignment of these regions show that all the aligned P22-like tail spikes contain conserved residues at these locations (including the Myovirus Det7), that set them apart from ΦSH19 Tsp3 (Figure 4C). The eight amino acids spanning residues 585-592 (V/G-G-P/A-G-S/T-G-S-A-W) retain a sequence motif similar to that found at the N-terminal boundary of ΦSH19 Tsp3, and the tail-associated proteins aligned in Figure 4A. However, this motif is absent at the C-terminal end of ΦSH19 Tsp3. P22-like tail spike modules may well have descended from a common ancestor with the flexibility to diverge their protein sequences whilst retaining the topology of the right-handed parallel β-helices. It has been proposed that this flexibility has allowed the protein domain to become widespread amongst bacteriophages [33], with the possibility of later domain interchange creating further diversity [38].

In summary, we have sequenced the genome of ΦSH19, a promising candidate for phage biosanitization of *S*. Typhimurium in the food production environment. The 157,785 bp circular dsDNA genome was found to contain no genes associated with toxicity or lysogeny, which is a pre-requisite for such applications. Analysis of the nucleotide sequence of ΦSH19 revealed only two close relatives in the database: *S*. Typhi-specific Vi01 and *Shigella*-specific SboM-AG3 that collectively form the Vi01-like phage family. Major differences were evident between ΦSH19 and Vi01 in three different tail spike proteins. Two tail spikes from ΦSH19 contain protein domains associated with the degradation of polysaccharides common to *Salmonella *LPS, namely the pectate lyase (Tsp2) and P22 tail spike-like (Tsp3) families of proteins. The acquisition of these domains is the most obvious reason to explain the different host specificities of ΦSH19, Vi01 (seemingly host-restricted due to the presence of a Vi antigen-degrading tail spike protein domain) and SboM-AG3 (whose tail spikes are as yet undefined). Based on amino acid sequence analysis the tail spikes from all three phages appear to form right-handed parallel β-helical structures. This appears to be an evolutionarily-conserved structure for all three tail spikes found in the Vi01-like phage. However, this conservation is coupled to the exchange of individual protein domains within these structures that may ultimately determine host range.

## Materials and methods

### Isolation of ΦSH19

For phage isolation, sewage effluent was filtered through 0.2 μm Minisart filters (Sartorius Biotech. Cat. No. 16534) and the filtrate collected in sterile universals and stored at 4°C until required. For *S*. Typhimurium lawn preparation NZCYM broth (Difco Cat. No. 240410) cultures were prepared and incubated overnight at 37°C with shaking. Following this, the overnight culture was used to seed fresh NZCYM broth containing 10 mM MgSO_4 _(Sigma Aldrich Chemicals Cat. No. M2643) which was then incubated for 2 hours at 37°C with shaking. To molten (tempered to ~ 50°C) NZCYM top agar containing 0.6% Bacteriological Agar No.1 (Oxoid Cat No. LP0011) 500 μL of the required *Salmonella *was added, followed by 500 μL filtrate, and the mixture was poured onto NZCYM agar plates. The plates were left to set on the bench for 20 minutes before being inverted then incubated overnight at 37°C. Any plaques identified were picked using sterile pipette tips and resuspended in 500 μL SM buffer (50 mM Tris-HCl [pH7.5], 100 mM NaCl, 8 mM MgSO_4_:7H_2_O, 0.01% gelatin, pH 7.5), incubated at 37°C for 1 hour, then serial diluted in SM buffer. A 25 μL volume of each dilution was then added to *Salmonella*/NZCYM top agar, and lawns were prepared as described above. This process was repeated three times in order to obtain a single clonal isolate of ΦSH19.

### Phage host range assays

The ability of phages ΦSH19 and Vi01 to lyse various *S*. Typhimurium serovars and *S*. Typhi BRD948 (kindly provided by D. Pickard Wellcome Trust Sanger Centre, UK) was determined as follows. *S*. Typhimurium and *S*. Typhi BRD948 top agar lawns were prepared as described above, with the exception of the latter being grown on supplemented minimal media as described by Tacket et al. [39]. To each bacterial lawn triplicate 20 μL volumes of log_10_7 PFU mL^-1 ^dilutions of the required phage were applied. Following a sufficient drying period, plates were inverted and incubated overnight at 37°C. The following day, each plate was observed for lysis and the results are shown in Table 1.

### Transmission electron microscopy

A freshly-prepared high titre phage suspension of ΦSH19 was sedimented at 34,900 × g for 2 hours (4°C). Following centrifugation, the supernatant was decanted and each phage pellet was washed twice with 0.1 M ammonium acetate for 1 hour at 25,000 × g. The wash solution was discarded and 2 mL SM buffer added to each centrifuge tube. Phage pellets were recovered following overnight incubation at 4°C with gentle shaking. A small drop of washed phage suspension was spotted onto a carbon-coated copper mesh grid and allowed to sit for 3 minutes. Excess phage suspension was then removed with filter paper. For negative staining one drop of phosphotungstic acid [pH 7.4] was added to each grid, and excess stain was removed one minute later with filter paper. Each grid was then covered and allowed to dry for 15 minutes. Images were taken with a Fei Tecnai Biotwin TEM (Fei Company, USA).

### ΦSH19 DNA extraction and sequencing

A high titre suspension of ΦSH19 (~ 10^10 ^PFU mL^-1^) was subjected to a single-step caesium chloride (CsCl) purification procedure as follows. CsCl (Melfords Cat. No. MB1006) was added to the high titre phage suspension to a final concentration of 0.75 g mL^-1^. Samples were then subjected to ultracentrifugation at 264,000 × g for 24 hours in a Beckman TL100 ultracentrifuge (15°C). Extraction of the band containing purified highly-concentrated ΦSH19 was then performed with a sterile 20-gauge hypodermic needle, and the band was subsequently collected in a sterile tube. Residual CsCl was removed from the sample using an Amicon^® ^Ultra-0.5 30 kDa MWCO centrifugal filter device (Millipore Cat. No. UFC 503008) as follows. Briefly, phage-CsCl solution was added to the column, which was then spun at 17,900 × g in a benchtop centrifuge for 30 mins. The column was then washed twice with SM buffer at 17,900 × g for 2 minutes. To elute the retained phage, SM buffer was added to the column which was then inverted and placed in a fresh collection tube. The column was then centrifuged at 17,900 × g for 10 minutes to recover the phage. ΦSH19 DNA was isolated from the purified stock using the phenol-chloroform extraction method with slight modifications as follows. An equal volume of 10 mg mL^-1 ^Proteinase K (Fisher Scientific Cat No. BPE 1700-500) was added to the CsCl-purified sample, followed by detergent solution (10 ng mL^-1 ^Proteinase K in 50 mM EDTA, 50 mM Tris-HCl [pH 8], 1% N-lauroyl sarcosine (Sigma Aldrich Chemicals, Cat No. L9150)). The solution was then incubated overnight at 55°C. Following this, phage DNA was extracted using phenol: chloroform and ethanol precipitation [5]. The extracted ΦSH19 DNA was purified using a DNA wizard purification kit (Promega, Cat No. A1120) then subjected to whole genome amplification using a Repli-G kit (Qiagen, UK Cat. No. 150023). Genomic DNA was fragmented to 500 bp using a Covaris S2 sonicator (Covaris Inc., USA) and libraries constructed using a NEBNext DNA Sample Prep Master Mix Set 2 (New England Biolabs Cat. No. E6070S). The libraries were subsequently sequenced using the Roche 454 GS FLX system (Roche Diagnostics, USA).

The fully sequenced ΦSH19 genome was annotated using a combination of CLC Genomics Workbench (CLC Bio, Denmark) and Artemis software [40]. For comparisons between the genomes of ΦSH19, Vi01, and SboM-AG3, ACT software was used [41], and comparison files were generated using the web-based programme DoubleACT http://www.hpa-bioinfotools.org.uk/pise/double_act.html. Nucleotide and protein searches were performed using the Blast search algorithm [42]. For the identification of protein domains of known function, searches of the Pfam database were made [43], whilst protein alignments were generated using ClustalW2 [44]. Betawrap was used to identify potential right-handed parallel β-helices in the tail spike proteins encoded by ΦSH19, Vi01, and SboM-AG3 [45]. For the identification of phage regulatory elements Phire analysis of the ΦSH19 genome was performed [46], and tRNAscan-SE 1.21 was used to identify tRNA genes [47]. The complete annotated ΦSH19 genome sequence has been deposited in the NCBI database (Genbank Acc. No. JN126049).

## Abbreviations

gp: gene product, used in the context of functional homologues to coliphage T4 proteins.

## Competing interests

The authors declare that they have no competing interests.

## Authors' contributions

SPTH and IFC contributed to the writing of this manuscript. SPTH contributed to all the experimental work reported here. ART contributed to the assembly and DNA sequence interpretation. JR and RW performed DNA sequencing. IFC was the principal investigator and provided all facilitates to complete this work. All authors approved the final manuscript.

## References

[B1] Mahony J McAuliffe O Ross RP van Sinderen D Bacteriophages as biocontrol agents of food pathogens Curr Opin Biotechnol 2011 22 157 163 10.1016/j.copbio.2010.10.008 21115341

[B2] Atterbury RJ Connerton PL Dodd CE Rees CE Connerton IF Application of host-specific bacteriophages to the surface of chicken skin leads to a reduction in recovery of Campylobacter jejuni Appl Environ Microbiol 2003 69 6302 6306 10.1128/AEM.69.10.6302-6306.2003 PMC201188 14532096

[B3] Wagenaar JA Van Bergen MA Mueller MA Wassenaar TM Carlton RM Phage therapy reduces Campylobacter jejuni colonization in broilers Vet Microbiol 2005 109 275 283 10.1016/j.vetmic.2005.06.002 16024187

[B4] Goode D Allen VM Barrow PA Reduction of experimental Salmonella and Campylobacter contamination of chicken skin by application of lytic bacteriophages Appl Environ Microbiol 2003 69 5032 5036 10.1128/AEM.69.8.5032-5036.2003 PMC169133 12902308

[B5] Loc Carrillo C Atterbury RJ el-Shibiny A Connerton PL Dillon E Scott A Connerton IF Bacteriophage therapy to reduce Campylobacter jejuni colonization of broiler chickens Appl Environ Microbiol 2005 71 6554 6563 10.1128/AEM.71.11.6554-6563.2005 PMC1287621 16269681

[B6] O'Flynn G Ross RP Fitzgerald GF Coffey A Evaluation of a cocktail of three bacteriophages for biocontrol of Escherichia coli O157:H7 Appl Environ Microbiol 2004 70 3417 3424 10.1128/AEM.70.6.3417-3424.2004 PMC427753 15184139

[B7] Abuladze T Li M Menetrez MY Dean T Senecal A Sulakvelidze A Bacteriophages reduce experimental contamination of hard surfaces, tomato, spinach, broccoli, and ground beef by Escherichia coli O157:H7 Appl Environ Microbiol 2008 74 6230 6238 10.1128/AEM.01465-08 PMC2570303 18723643

[B8] Jamalludeen N Johnson RP Shewen PE Gyles CL Evaluation of bacteriophages for prevention and treatment of diarrhea due to experimental enterotoxigenic Escherichia coli O149 infection of pigs Vet Microbiol 2009 136 135 141 10.1016/j.vetmic.2008.10.021 19058927

[B9] Atterbury RJ Van Bergen MA Ortiz F Lovell MA Harris JA De Boer A Wagenaar JA Allen VM Barrow PA Bacteriophage therapy to reduce salmonella colonization of broiler chickens Appl Environ Microbiol 2007 73 4543 4549 10.1128/AEM.00049-07 PMC1932804 17526794

[B10] Wall SK Zhang J Rostagno MH Ebner PD Phage therapy to reduce preprocessing Salmonella infections in market-weight swine Appl Environ Microbiol 2010 76 48 53 10.1128/AEM.00785-09 PMC2798657 19854929

[B11] United States Food and Drug Administration http://www.fda.gov/OHRMS/DOCKETS/98fr/cf0559.pdf

[B12] Carlton RM Noordman WH Biswas B de Meester ED Loessner MJ Bacteriophage P100 for control of Listeria monocytogenes in foods: genome sequence, bioinformatic analyses, oral toxicity study, and application Regul Toxicol Pharmacol 2005 43 301 312 10.1016/j.yrtph.2005.08.005 16188359

[B13] Guenther S Huwyler D Richard S Loessner MJ Virulent bacteriophage for efficient biocontrol of Listeria monocytogenes in ready-to-eat foods Appl Environ Microbiol 2009 75 93 100 10.1128/AEM.01711-08 PMC2612219 19011076

[B14] Soni KA Nannapaneni R Removal of Listeria monocytogenes biofilms with bacteriophage P100 J Food Prot 2010 73 1519 1524 10.4315/0362-028x-73.8.1519 20819365

[B15] Soni KA Nannapaneni R Hagens S Reduction of Listeria monocytogenes on the surface of fresh channel catfish fillets by bacteriophage Listex P100 Foodborne Pathog Dis 2010 7 427 434 10.1089/fpd.2009.0432 19958102

[B16] Merabishvili M Pirnay JP Verbeken G Chanishvili N Tediashvili M Lashkhi N Glonti T Krylov V Mast J Van Parys L Lavigne R Volckaert G Matthews W Verween G De Corte P Rose T Jennes S Zizi M De Vos D Vaneechoute M Quality-controlled small-scale production of a well-defined bacteriophage cocktail for use in human clinical trials PLoS One 2009 4 e4944 10.1371/journal.pone.0004944 PMC2654153 19300511

[B17] Gill JJ Hyman P Phage choice, isolation, and preparation for phage therapy Curr Pharm Biotechnol 2010 11 2 14 10.2174/138920110790725311 20214604

[B18] Herold S Karch H Schmidt H Shiga toxin-encoding bacteriophages--genomes in motion Int J Med Microbiol 2004 294 115 121 10.1016/j.ijmm.2004.06.023 15493821

[B19] Allison HE Stx-phages: drivers and mediators of the evolution of STEC and STEC-like pathogens Future Microbiol 2007 2 165 174 10.2217/17460913.2.2.165 17661653

[B20] Wagner PL Waldor MK Bacteriophage control of bacterial virulence Infect Immun 2002 70 3985 3993 10.1128/IAI.70.8.3985-3993.2002 PMC128183 12117903

[B21] Salmonella in livestock production in GB 2009 http://vla.defra.gov.uk/reports/docs/rep_salm09_chp4.pdf

[B22] Pickard D Toribio AL Petty NK van Tonder A Yu L Goulding D Barrell B Rance R Harris D Wetter M Wain J Choudhary J Thomson N Dougan G A conserved acetyl esterase domain targets diverse bacteriophages to the Vi capsular receptor of Salmonella enterica serovar Typhi J Bacteriol 2010 192 5746 5754 10.1128/JB.00659-10 PMC2953684 20817773

[B23] Anany H Lingohr EJ Villegas A Ackermann HW She YM Griffiths MW Kropinski AM A Shigella boydii bacteriophage which resembles Salmonella phage ViI Virol J 2011 8 242 10.1186/1743-422X-8-242 PMC3121705 21595934

[B24] Hooton SP Atterbury RJ Connerton IF Application of a bacteriophage cocktail to reduce Salmonella Typhimurium U288 contamination on pig skin Int J Food Microbiol in press 10.1016/j.ijfoodmicro.2011.08.015 21899907

[B25] De Lappe N Doran G O'Connor J O'Hare C Cormican M Characterization of bacteriophages used in the Salmonella enterica serovar Enteritidis phage-typing scheme J Med Microbiol 2009 58 86 93 10.1099/jmm.0.000034-0 19074657

[B26] Steinbacher S Baxa U Miller S Weintraub A Seckler R Huber R Crystal structure of phage P22 tailspike protein complexed with Salmonella sp. O-antigen receptors Proc Natl Acad Sci USA 1996 93 10584 10588 10.1073/pnas.93.20.10584 PMC38196 8855221

[B27] Walter M Fiedler C Grassl R Biebl M Rachel R Hermo-Parrado XL Llamas-Saiz AL Seckler R Miller S van Raaij MJ Structure of the receptor-binding protein of bacteriophage det7: a podoviral tail spike in a myovirus J Virol 2008 82 2265 2273 10.1128/JVI.01641-07 PMC2258939 18077713

[B28] Yoder MD Keen NT Jurnak F New domain motif: the structure of pectate lyase C, a secreted plant virulence factor Science 1993 260 1503 1507 10.1126/science.8502994 8502994

[B29] Mayans O Scott M Connerton I Gravesen T Benen J Visser J Pickersgill R Jenkins J Two crystal structures of pectin lyase A from Aspergillus reveal a pH driven conformational change and striking divergence in the substrate-binding clefts of pectin and pectate lyases Structure 1997 5 677 689 10.1016/s0969-2126(97)00222-0 9195887

[B30] Jenkins J Mayans O Pickersgill R Structure and evolution of parallel beta-helix proteins J Struct Biol 1998 122 236 246 10.1006/jsbi.1998.3985 9724625

[B31] Steinbacher S Seckler R Miller S Steipe B Huber R Reinemer P Crystal structure of P22 tailspike protein: interdigitated subunits in a thermostable trimer Science 1994 265 383 386 10.1126/science.8023158 8023158

[B32] Muller JJ Barbirz S Heinle K Freiberg A Seckler R Heinemann U An intersubunit active site between supercoiled parallel beta helices in the trimeric tailspike endorhamnosidase of Shigella flexneri Phage Sf6 Structure 2008 16 766 775 10.1016/j.str.2008.01.019 18462681

[B33] Barbirz S Muller JJ Uetrecht C Clark AJ Heinemann U Seckler R Crystal structure of Escherichia coli phage HK620 tailspike: podoviral tailspike endoglycosidase modules are evolutionarily related Mol Microbiol 2008 69 303 316 10.1111/j.1365-2958.2008.06311.x 18547389

[B34] Thompson JE Pourhossein M Waterhouse A Hudson T Goldrick M Derrick JP Roberts IS The K5 lyase KflA combines a viral tail spike structure with a bacterial polysaccharide lyase mechanism J Biol Chem 2010 285 23963 23969 10.1074/jbc.M110.127571 PMC2911314 20519506

[B35] Wollin R Eriksson U Lindberg AA Salmonella bacteriophage glycanases: endorhamnosidase activity of bacteriophages P27, 9NA, and KB1 J Virol 1981 38 1025 1033 10.1128/jvi.38.3.1025-1033.1981 PMC171242 7017163

[B36] Weigele PR Scanlon E King J Homotrimeric, beta-stranded viral adhesins and tail proteins J Bacteriol 2003 185 4022 4030 10.1128/JB.185.14.4022-4030.2003 PMC164894 12837775

[B37] Andres D Baxa U Hanke C Seckler R Barbirz S Carbohydrate binding of Salmonella phage P22 tailspike protein and its role during host cell infection Biochem Soc Trans 2010 38 1386 1389 10.1042/BST0381386 20863318

[B38] Casjens SR Thuman-Commike PA Evolution of mosaically related tailed bacteriophage genomes seen through the lens of phage P22 virion assembly Virology 2011 411 393 415 10.1016/j.virol.2010.12.046 21310457

[B39] Tacket CO Sztein MB Losonsky GA Wasserman SS Nataro JP Edelman R Pickard D Dougan G Chatfield SN Levine MM Safety of live oral Salmonella typhi vaccine strains with deletions in htrA and aroC aroD and immune response in humans Infect Immun 1997 65 452 456 10.1128/iai.65.2.452-456.1997 PMC174616 9009296

[B40] Rutherford K Parkhill J Crook J Horsnell T Rice P Rajandream MA Barrell B Artemis: sequence visualization and annotation Bioinformatics 2000 16 944 945 10.1093/bioinformatics/16.10.944 11120685

[B41] Carver TJ Rutherford KM Berriman M Rajandream MA Barrell BG Parkhill J ACT: the Artemis Comparison Tool Bioinformatics 2005 21 3422 3423 10.1093/bioinformatics/bti553 15976072

[B42] Altschul SF Gish W Miller W Myers EW Lipman DJ Basic local alignment search tool J Mol Biol 1990 215 403 410 10.1016/S0022-2836(05)80360-2 2231712

[B43] Bateman A Coin L Durbin R Finn RD Hollich V Griffiths-Jones S Khanna A Marshall M Moxon S Sonnhammer EL Studholme DJ Yeats C Eddy SR The Pfam protein families database Nucleic Acids Res 2004 32 D138 141 10.1093/nar/gkh121 PMC308855 14681378

[B44] Larkin MA Blackshields G Brown NP Chenna R McGettigan PA McWilliam H Valentin F Wallace IM Wilm A Lopez R Thompson JD Gibson TJ Higgins DC Clustal W and Clustal × version 2.0 Bioinformatics 2007 23 2947 2948 10.1093/bioinformatics/btm404 17846036

[B45] Bradley P Cowen L Menke M King J Berger B BETAWRAP: successful prediction of parallel beta -helices from primary sequence reveals an association with many microbial pathogens Proc Natl Acad Sci USA 2001 98 14819 14824 10.1073/pnas.251267298 PMC64942 11752429

[B46] Lavigne R Sun WD Volckaert G PHIRE, a deterministic approach to reveal regulatory elements in bacteriophage genomes Bioinformatics 2004 20 629 635 10.1093/bioinformatics/btg456 15033869

[B47] Schattner P Brooks AN Lowe TM The tRNAscan-SE, snoscan and snoGPS web servers for the detection of tRNAs and snoRNAs Nucleic Acids Res 2005 33 W686 689 10.1093/nar/gki366 PMC1160127 15980563

